# Unearthing how, why, for whom and under what health system conditions the antiretroviral treatment adherence club intervention in South Africa works: A realist theory refining approach

**DOI:** 10.1186/s12913-018-3150-6

**Published:** 2018-05-09

**Authors:** Ferdinand C. Mukumbang, Bruno Marchal, Sara Van Belle, Brian van Wyk

**Affiliations:** 1 School of Public Health, University of the Western Cape, Cape Town, South Africa; 2 Department of Public Health, Institute of Tropical Medicine, Antwerp, Belgium

**Keywords:** Adherence, Adherence club, Antiretroviral therapy, Configurational mapping. Intervention-context-Actor-mechanism-outcome configuration, Generative mechanisms, Programme theory, Realist evaluation, Retention in care, Retroduction

## Abstract

**Background:**

Poor retention in care and suboptimal adherence to antiretroviral treatment (ART) undermine its successful rollout in South Africa. The adherence club intervention was designed as an adherence-enhancing intervention to enhance the retention in care of patients on ART and their adherence to medication. Although empirical evidence suggests the effective superiority of the adherence club intervention to standard clinic ART care schemes, it is poorly understood exactly how and why it works, and under what health system contexts. To this end, we aimed to develop a refined programme theory explicating how, why, for whom and under what health system contexts the adherence club intervention works (or not).

**Methods:**

We undertook a realist evaluation study to uncover the programme theory of the adherence club intervention. We elicited an initial programme theory of the adherence club intervention and tested the initial programme theory in three contrastive sites. Using a cross-case analysis approach, we delineated the conceptualisation of the intervention, context, actor and mechanism components of the three contrastive cases to explain the outcomes of the adherence club intervention, guided by retroductive inferencing.

**Results:**

We found that an intervention that groups clinically stable patients on ART in a convenient space to receive a quick and uninterrupted supply of medication, health talks, counselling, and immediate access to a clinician when required works because patients’ self-efficacy improves and they become motivated and nudged to remain in care and adhere to medication. The successful implementation and rollout of the adherence club intervention are contingent on the separation of the adherence club programme from other patients who are HIV-negative. In addition, there should be available convenient space for the adherence club meetings, continuous support of the adherence club facilitators by clinicians and buy-in from the health workers at the health-care facility and the community.

**Conclusion:**

Understanding what aspects of antiretroviral club intervention works, for what sections of the patient population, and under which community and health systems contexts, could inform guidelines for effective implementation in different contexts and scaling up of the intervention to improve population-level ART adherence.

## Background

South Africa has the largest HIV-treatment programme in the world, accounting for 20% of people on antiretroviral therapy globally [1]. There is growing evidence that differentiated care models employed in the management of HIV have the potential to improve and sustain adherence to medication and retention in care of people living with HIV [2, 3]. With an estimated 7.1 million people living with HIV in South Africa as at 2017 [4], differentiated care models have been advanced for the management of very large HIV-patient cohorts at the primary health-care level.

The adherence club intervention [5–7] a type of a differentiated care model, is implemented in Western Cape Province, South Africa to address challenges of poor retention in care – attending scheduled clinical visits – and suboptimal adherence to ART – taking medication as prescribed. The adherence club intervention is an ancillary ART service delivery model designed to streamline ART delivery for stable adult (18+ years), treatment-experienced patients, with good clinic attendance records and good medication adherence (evidenced by the two most recent consecutive viral loads undetectable (< 400 copies/mL)). Through group consultations, convenient medication pick-up processes, facilitated access to a clinician when needed, the adherence club provides ART patients with the required clinic care and drastically reduces their waiting times [5–7].

The adherence club programme shows potential to relieve clinic congestion and improve retention in care and treatment adherence in the context of the rapidly growing HIV-patient populations on ART [3, 8–10]. Although empirical evidence suggests that the adherence club model is more effective in retaining people living with HIV on ART and sustaining medication adherence compared to standard clinic care, it is poorly understood exactly how and why it works. To this end, a realist evaluation was proposed [6]. In this article, we report on the process of synthesising the findings obtained from three case studies to formulate a refined theory of which intervention modalities of the adherence club intervention work, for whom, in what circumstances, in what respects and why?

### Methodological approach

**Fig. 1:**
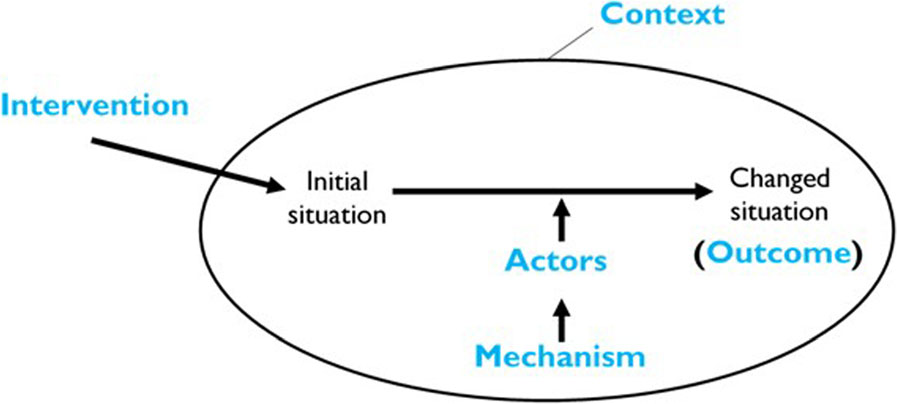
A generative configuration of realist theories

Realist evaluation is a theory-driven approach to programme evaluation [11], which strives to answer the question what works, for whom and under what circumstances [12, 13] to explain how and why programmes, policies and interventions work (or not). For an intervention to work, it must influence the reasoning (mechanism) of the targeted actors to cause them to adopt an intended behaviour that in a specific context will lead to a specific outcome. Therefore, realists assume that an outcome (O) is generated by a mechanism (M) being triggered in a particular context (C) through an actor (A) when an intervention (I) is implemented (Fig. 1). This conceptualisation captures how, why, for whom and in what circumstances programmes work. Formulating realist theories is, therefore, achieved through the formation of Intervention-context-actor-mechanism-outcome (ICAMO) configurations [14].

**Fig. 2:**
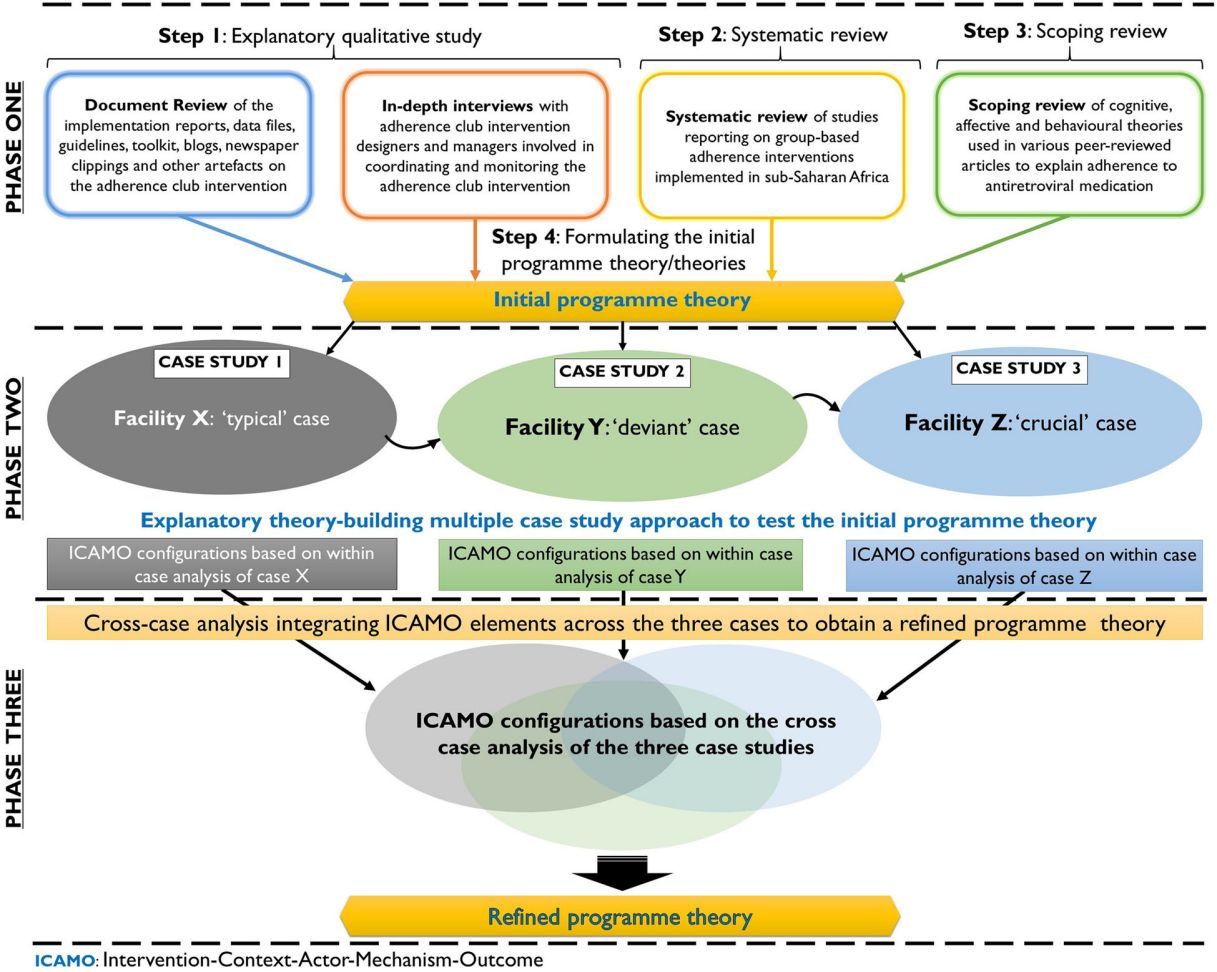
Three phases of realist evaluation inquiry

Realist evaluators go into the evaluation process with some expectations, guided by the initial programme theory. During the evaluation, some expectations are confirmed, and some might prove to be misguided. The end product of the analysis is expected to improve the picture of the programme efficacy and inefficacy [15]. Therefore, three principal phases are identified when conducting a realist evaluation inquiry. 1) Eliciting the initial programme theory; 2) testing the initial programme theory in contrastive sites, and 3) building a more refined programme theory based on the findings from the contrastive case studies (Fig. 2).

In the first phase of this work, we formulated the initial programme theory of the adherence club intervention [16]. We first conducted an exploratory qualitative study of programme designers’ and managers’ assumptions and perspectives of the intervention and carried out a document review of the design, rollout, implementation and outcome of the adherence clubs [17]. We also conducted a systematic review of available studies on group-based ART adherence support models in sub-Saharan Africa to tease out their underlining theories [18]. In addition, we carried out a scoping review of social, cognitive and behavioural theories that have been applied to explain adherence to ART [19].

Using the process of configuration mapping, we constructed an ICAMO map representing the initial programme theory of the adherence club through the process of retroduction – mechanism centred logic and analysis. Finally, we used the “if…then…because” statements to translate the ICAMO configuration map into testable hypothesis [20] (Table 1). Formulating the initial programme theory signifies the end of the first phase of the evaluation.

**Table 1:** Initial programme theory of the adherence club intervention

Initial programme theory	If…then…Because statement
Initial Programme Theory 1	IF adult (18+ years) clinically ‘stable’ patients with evidence of good clinic attendance are group-managed, receive quick symptom checks, quick access to medication, consistent counselling and social support from the peer counsellor, THEN patients are likely to adhere to medication and remain in care, BECAUSE they develop a group identity, which improves their perceived social, support, satisfaction and trust; and acquire knowledge, which helps them to understand their perceived threat and perceived benefits and improves their self-efficacy. As a result, they become encouraged, empowered and motivated, thus, more likely to remain in care and adhere to the treatment.
Initial Programme Theory 2	IF operational staff receive goals and targets set to continuously enrol patients in the adherence club and strictly monitor their participation through strict standard operating practices (the promise of exclusion in the event of missed appointment and active patient tracing), THEN patients are likely to adhere to medication and remain in care, BECAUSE they fear (perceived fear) losing the benefits (easy access to medication, peer support, reduced waiting times, and two-month ART collection) of the club system and they are coerced through adhesive club rules. As a result, they become nudged to remain in care and adhere to the treatment, which might decongest the health facility.

In the second phase, we applied an explanatory theory-building multiple case study approach [21] to test the initial programme theory in three contrastive sites described as typical, deviant or crucial [22]. Each site or facility was considered a unit of analysis, and each pair of adherence clubs being sub-units, which are embedded in the cases. Within each case, we tested the initial programme theory for its adequacy. We obtained modified versions of the initial programme theory explicating how and why the adherence club intervention works within that case (or not).

The third phase involves refining the programme theory based on the theories developed from the case studies, which is the focus of this paper. Therefore, in this paper, we report on the cross-case analysis towards developing a more refined programme theory of how and why the adherence club intervention works and under what health systems context.

### Ethical approval

This study is part of a larger project “A realist evaluation of the antiretroviral treatment adherence club programme in selected primary health care facilities in the metropolitan area of Western Cape Province, South Africa”, which has received ethics clearance from the Higher Degree’s committee of the University of the Western Cape (Reg No: 15/6/28). In addition, we obtained ethical clearance from the Provincial Department of Health of the Western Cape Province for the health facilities included.

### Study design

In this study, we applied a cross-case study design. The cross-case analysis is a research method that facilitates the comparison of commonalities and difference in the events, activities and processes through identified units of analyses [23]. Thus, it enabled us to delineate the combination of the intervention, context and mechanism components to explain the outcomes of the adherence club intervention. Using this method helped us to construct an explanation as to why one case was different or the same as others and to examine further the initial programme theory.

The confirmatory theory testing approach was applied. This approach uses predominantly hypothetico-deductive forms of reasoning, which entails moving from a theoretical concept (initial programme theory) to empirical testing of hypotheses [24]. In this approach, the researcher enters the research situation with an a priori theory and the purpose of the data collection is to ‘confirm’ or ‘disconfirm’ or modify the theory.

### Case selection

A maximum variation case selection approach was adopted [21]. This is an exploratory sampling strategy to identify the typical cases that are subsequently selected. Our aim of selecting cases with varying characteristics was to predict contrasting results to increase the degree of certainty of the results. Our case selection was, therefore, purposive and based on the 2014 routine data on the retention in care rates of clinics in the Western Cape Province. We classified ‘good’ retention in care as values above 80%. A value between 70% and 80% was considered ‘average’ and any value below 70% was identified as ‘poor’ retention in care. Case X had a retention in care rate of 70%, Case Y, 63.0% and Case Z, 81.7%. Based on these values, we labelled our cases as typical, deviant and crucial, respectively [22]. In Table 2**,** the characteristics of the facilities selected for the case studies in 2014 are shown.

**Table 2:** Characteristics of facilities selected for case studies

Characteristics	Case X ‘Typical’	Case Y ‘Deviant’	Case Z ‘Crucial’
Adult patients on ARVs in August 2014	2561	1501	1486
Number of ACs	39	2	20
Official starting date of AC	2012	2014	2012
Number of patients in adherence club care	1309	35	480
Number of ART staff	11	09	08
Implementation context	rollout	rollout	rollout
Predominant catchment population	Coloured	Black	Coloured

### Data congregation

Three case studies were conducted. Within each case, we used quantitative data to identify and classify the outcome patterns and qualitative data to explore implementation features related to the context (observation) and the mechanism (in-depth interviews). We thus combined a retrospective cohort analysis and an explanatory qualitative approach. The retrospective cohort analysis was conducted to describe the primary outcomes of the adherence club intervention (retention in care and adherence to medication) and the qualitative explanatory design provided evidence regarding the ICAMO configuration links in the implementation chain (intervention modalities, actors involved, generative mechanisms, relevant context and outcome patterns).

Regarding the quantitative study, we purposively selected two clubs among the number of clubs that were opened and reached the maximum capacity (35–45) in 2012. First, we identified the clubs that had reached the maximum capacity. Then, from the number that had reached maximum number, we used the fishbowl method of sampling to obtain two adherence clubs to be included in the analysis.

For the interviews, we adopted the purposive sampling technique - based on the participant’s potential contributions toward clarifying the initial programme theory. It is suggested by Pawson and Tilley [13] that practitioners have specific ideas on what it is within the programme that works (M), knowledge on the outcomes (O) of the programme (because they are likely to have experienced successes and failures) and some awareness of people and places (C) for whom the programme works. On the other side, patients are more likely to be sensitised about mechanisms and intervention modalities than to its contextual constraints and outcome patterns [13].

#### Case study 1: Case X

Case X, where the initial programme theory was tested first, represents a ‘typical’ case regarding the implementation of the adherence club because it was among the facilities recruited for first phase rollout in 2012. Since its inception, the adherence club programme at Case X facility has shown steady growth at a reasonable pace.

At this facility, the adherence club programme operates in a separate Unit within the main clinic. The dedicated staff consist of receptionists (2), a data clerk (1), counsellors (3), nurses (3) and doctors (used to be two in 2014 but one since 2016). The adherence club programme grew steadily with no noticeable disruptions regarding the organisation and operation. The adherence club Unit has offices allocated to all the above-mentioned staff including a big area dedicated to the adherence club activities. With all the necessary facilities available and with buy-in from the health-care providers, the adherence club programme progressed steadily and was identified through the theory obtained after testing the initial programme theory as a positive case.

The ICAMO configurations obtained (Table 3) are based on qualitative and quantitative data collected to identify and describe the mechanism, context, actors and outcome.

**Table 3:** Intervention-Context-Actor-Mechanism-Outcome configurations of Case X

Intervention modalities	Context	Actor	Mechanism	Outcome
Club rules and regulation	- Standard operating protocol - HIV policy	- Patient	- Perceived barriers - Perceived threat - Nudged	- Adhering to club appointments
Grouping patients	- Availability of space for meeting - Longevity of patient in the club - Relationship with other club members	- Patient - Group	- Perceived social support - Positive group dynamics (sense of belonging)	- Better adherence resulting from developed self-efficacy
Health talks/education	- Availability of personnel	- Patient	- Empowerment (motivation)	- Improved self-efficacy
Quick medication access	- Availability of medication - Proper preparation for club session	- Patient	- Perceived benefit - Motivation - Satisfaction	- Adherence to medication related to medication access
Prompt continuity of care	- Availability of clinicians - Staffing dynamics - Organisation of club activities	- Clinicians - Patient	- Trust	- Retained in care through problem resolution
Club facilitator-patient relationship	- Staffing dynamics - Teamwork/collaboration	- Facilitator - Patient	- Trust - Perceived support	- Adherence to medication - Retention in care
Overall intervention	- Buy-in from care providers - Preparation and organisation	- Patients - Club teams	- Motivation - Self-efficacy - Satisfaction (with care)	- Improved retention in care and adherence to medication

#### Case study 2: Case Y

Case Y was identified as a ‘deviant’ case for two reasons. First, the retention in care rates of patients on ART at this facility was very low compared to the other facilities in the district. Second, while the initial implementation of the adherence club programme at the facility was scheduled for the first phase rollout in 2012 along with the other facilities, adherence clubs were only implemented officially in 2014. Two factors were responsible for the delayed implementation of the programme at the facility; these were lack of a ‘dedicated venue’ as meeting place for the club members and adherence counselling offices and poor buy-in from the facility health-care workers. Regarding staffing, this facility had a dedicated club doctor, two nurses, three counsellors and an admin clerk and data capture clerk. The counsellors also counselled patients with TB.

In 2015, a nurse was identified from another facility and trained in the various aspects of the implantation of the adherence club programme. She championed the implementation of the programme by heading the coordination of the club activities and educating the other health-care providers to get their buy-in. Once these two components were secured, the adherence club programme in facility Y started performing well and was identified, through the data analysis as a positive case – confirming the initial programme theory (Table 4).

**Table 4:** Intervention-Context-Actor-Mechanism-Outcome configurations of Case Y

Intervention modalities	Context	Actor	Mechanism	Outcome
Club rules and regulation	- Standard operating protocol - Being reminded of the rules and regulations of the club - HIV policy	- Patient	- Perceived barriers - Perceived coercion - Perceived fear - Reinforcement - nudged	- Nudged to adhere to club appointments
Group dynamics	- Availability of space for meeting - Longevity of patient in the club - Relationship with other club members	- Patient - Group	- Perceived social support - Bonding and formation of group identity	- Better adherence resulting from developed self-efficacy
Health talks/education	- Availability of personnel - Team work	- Patient	- Motivation - Empowerment	- Improved self-efficacy
Quick medication access	- Availability of medication - Organisation of pick-up process and club sessions - Buy-in from care providers	- Patient	- Perceived benefit - Motivation - Satisfaction	- Adherence to medication related to medication availability
Prompt continuity of care	- Availability of clinicians - Staffing dynamics - Organisation of club activities - Buy-in from care providers	- Clinicians - Patient	- Trust - Satisfaction	- Retained in care through problem resolution
Club facilitator-patient relationship	- Staffing dynamics - Teamwork/collaboration - Buy-in from care providers	- Facilitator - Patient	- Trust - Perceived support	- Adherence to medication - Retention in care
Overall intervention	- Availability of programme champion - Buy-in from care providers - Preparation and organisation	- Patients - Club teams	- Motivation - Self-efficacy - Satisfaction	- Improved retention in care and adherence to medication

#### Case study 3: Case Z

Case Z was considered a ‘critical’ case because, at the time of case selection (2014), the facility showed good retention in care rates for their overall ART programme. Following the successes of the adherence club programme and the overall ART programme at Case Z facility, this site was proposed for piloting the integration of chronic care in 2015. This meant that the ART services had to move back to the main clinic to be situated where the other chronic conditions such as diabetes, hypertension, arthritis and epilepsy were managed. Although the ART patients and those managed for chronic care were being managed within the same Unit, separate teams of health-care providers managed them using separate treatment management schedules and treatment strategies. Regarding staffing, the facility had a dedicated doctor, two nurses, two counsellors, and a data capture clerk. Nevertheless, the facility faced challenges of lack of space, inconvenience and exposure to inadvertent disclosure when managing ART patients resurfaced, so the management decided to move the ‘integrated’ services back to the separate building in 2016 where the services currently operate.

Although ART and other chronic care services are organised within the same Unit, they are coordinated separately. On one side of the building the non-chronic care services are conducted, and on the other side the adherence club programme, but the patients share a common waiting area. Therefore, the building that was once dedicated exclusively to ART services is being shared with the other chronic care services Unit; thus, lack of space and confidentiality problems are the prevailing conditions. In Table 5**,** the ICAMO matrix obtained after data analysis at case Z facility is shown.

**Table 5:** Intervention-Context-Actor-Mechanism-Outcome configurations: Case Z

Intervention modalities	Context	Actor	Mechanism	Outcome
Club rules and regulation	- Integration of HIV treatment with other chronic diseases of lifestyle - Unconducive environment - Lack of resources - Presence of non-HIV positive patients	- Patient	- Perceived stigma - Poor knowledge of club rules and regulations - Perceived absence of punitive measures	- Inadvertent disclosure of HIV status - Poor attendance at club appointments
Group dynamics	- Unconducive environment - Lack of resources - Experimenting various execution models	- Patient - Group	- Perceived lack of social support - Feeling of frustration related to loss of group dynamics	- Reduced adherence related to constant changes and disruptions in group dynamics
Health talks/education	- Lack of resources - Presence of non-HIV positive patients - Unconducive environment	- Patient	- Perceived inadequacy	- Reduced self-efficacy leading to poor retention in care and medication adherence - Inadvertent disclosure of HIV status
Quick medication access	- Unconducive environment - Lack of resources - Experimenting various execution models	- Patient	- Perceived benefit - Perceived stigma	- Adherence to medication related to medication availability - Poor adherence resulting from poor club attendance
Prompt continuity of care	- Poor adherence club programme coordination and execution	- Clinicians - Patient	- Role confusion - Dissatisfaction	- Reduced rate of retention in care
Club facilitator-patient relationship	- Unconducive environment - Lack of health talks and counselling sessions	- Facilitator - Patient	- Trust - Perceived lack of support	- Poor adherence to medication - Poor retention in care
Overall intervention	- Unconducive environment - Lack of resources - Experimenting various execution models - Presence of non-HIV positive patients - Experimenting various execution models - Poor adherence club programme coordination	- Patients - Club teams	- Demotivation - Frustration - Confusion	- Reduced attendance at club sessions - Poor retention in care - Reduced medication adherence rates

### Data analysis

The process of synthesising the findings of the three case studies followed a confirmatory theory building approach. Confirmatory theory building approaches, especially when applied within realist studies, use a retroductive (or abductive) form of reasoning as the central approach to inference making [24]. This allowed us to move from descriptions of the concrete to the abstract, and back to the concrete [13]. After obtaining the modified programme theories from the three contrastive sites, we applied an analytical process that involved the identification of ICAMO components across the three cases and linked them to formulate a refined theory of the initial programme theory. This process involved the application of various analytic techniques, retroduction (mechanism-centred theorising), counterfactual thinking (comparison of theories) and abstractions (analytical generalisation), and concretisation of the theorised mechanisms in different situations of configurational thinking.

**Fig. 3:**
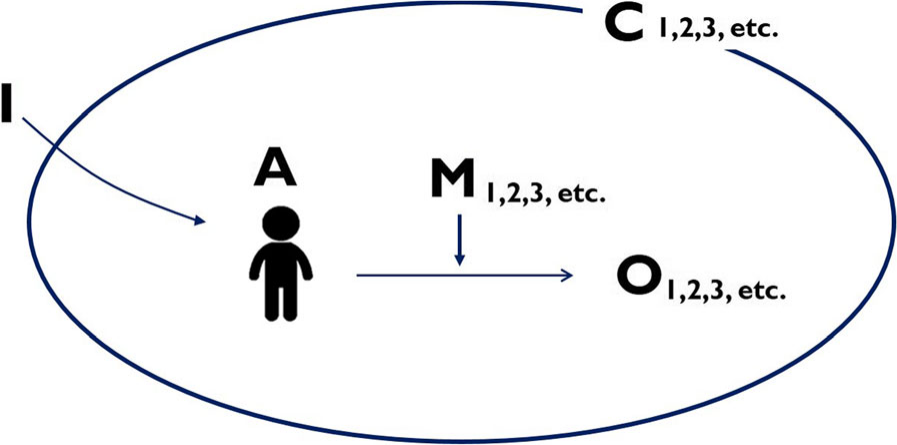
Comparing various contexts to develop a refined ICAMO configuration

Our analysis was guided by retroductive inferencing within the configurational mapping – a logic in which outcomes are considered to follow from the alignment, within a case, of a specific combination of attributes – of the elements of the realist heuristic tool [25]. The conjectured ICAMO configurations, of each of the three cases, were compared and contrasted in the search for general models (Fig. 3).

Comparison and contrasting were done by linking each active mechanism identified as being associated with a positive outcome (M-O links), then we looked for the context in which the mechanism was contingent. The ICAMO prototype from the negative case was used to adjust (confirm) certain links to develop ICAMOs based on failed outcome scenarios, and as a reason to abandon certain ICAMO configuration chains [26]. According to Mingers [27] the interplay between positive or counteracting mechanisms determines whether events occur or not. Comparing the negative and positive cases also provided evidence for adjusting the ICAMO configurations as in some cases; the negative ones enforced the construction of positive ones. This was achieved by identifying the association of the failed outcomes with ‘missing mechanisms’ and ‘negative contexts’. The process required the application of counterfactual thinking (testing possible alternative explanations) to argue towards *transfactual* (mechanism-centred) conditions [24]. In applying this counterfactual [and transfactual] thinking, we constructed ICAMO maps (Figs. 5, 6, 7, 8, 9 and 10) of each of the modalities of the adherence club intervention based on the ICAMO heuristic tool to obtain a configurational causality representation of each intervention modality.

**Fig. 4:**
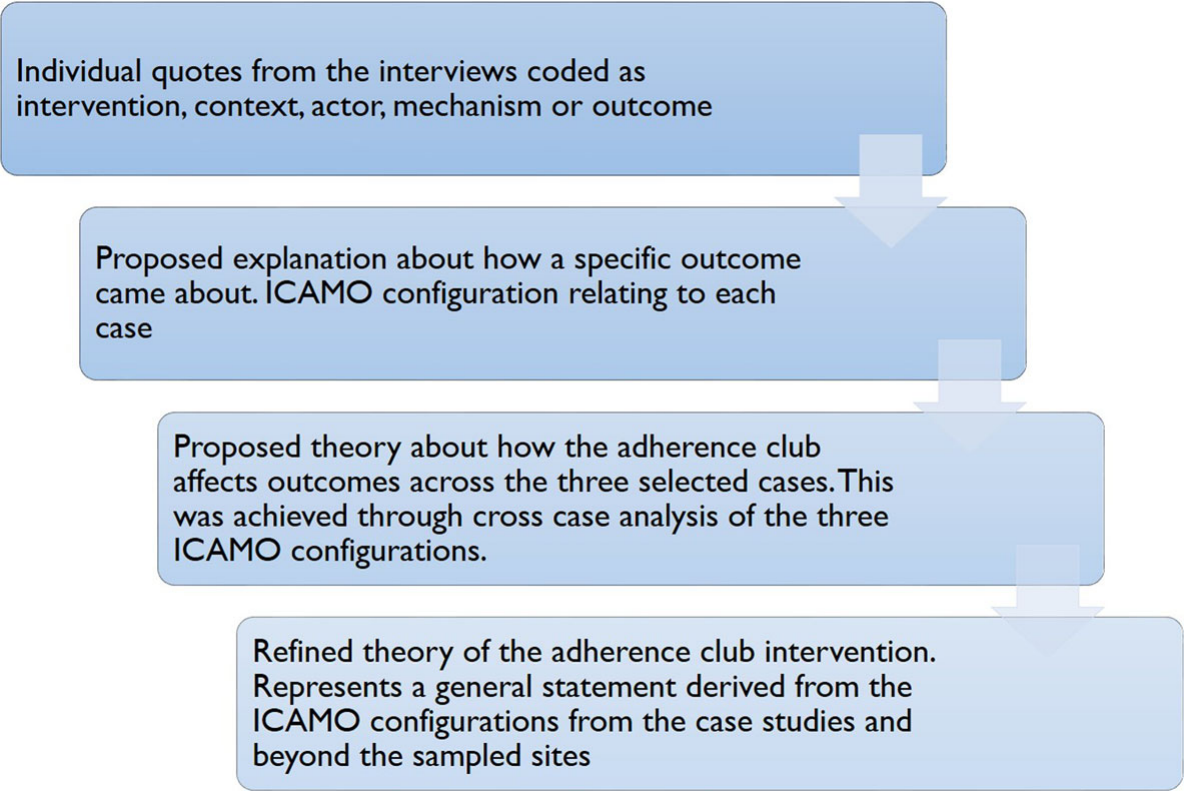
Application of the analytical generalisation to refine programme theory

The process of moving from the specifics of individual cases to a theory that is more abstract is known as the analytical generalisation and is outlined in Fig. 4.

## Results

Our analysis followed the five modalities associated with the adherence club intervention, i.e. the club rules and regulations, the grouping of the patients, quick medication pick-up, prompt continuity of care, facilitator-patient relationship and the overall adherence club intervention. After refining the ICAMO configurations related to each of the adherence club intervention modalities, we used the ‘**if…then…because**’ phraseology to explain the conceptualisation of the configurations. The ‘if…then…because’ phraseology follows that: ***if*** certain resources (information, material, opportunities, and sometimes constraints) are provided, ***then*** they will trigger the actors reasoning to a sufficient extent that a change to healthier behaviour will follow [15].

### Club rules and regulations

The adherence club intervention has rules and regulations that govern its functioning. During the introductory visit to the club, patients are provided with the rules and regulations governing the adherence club and its activities and reminded of these on every adherence club visit. For this intervention to work, users (patients) are meant to abide by the rules and regulations of the adherence club. In this way, the rules and regulations introduce a new set of mechanism(s) within the social context of the ART programme.

According to the club rules and regulations, club membership can be terminated if the patient has a viral load above 400 copies/mL or significantly abnormally low CD4 count < 200 cells/mm^3^, and when they develop an active TB infection. These are considered as proxies for non-adherence to medication. When a patient fails to attend mandatory club sessions regularly or when s/he fails to send a ‘treatment buddy’ to collect their medication from the club facilitator or club nurse within 5 days (grace period), they are also returned to the main clinic care.

**Fig. 5:**
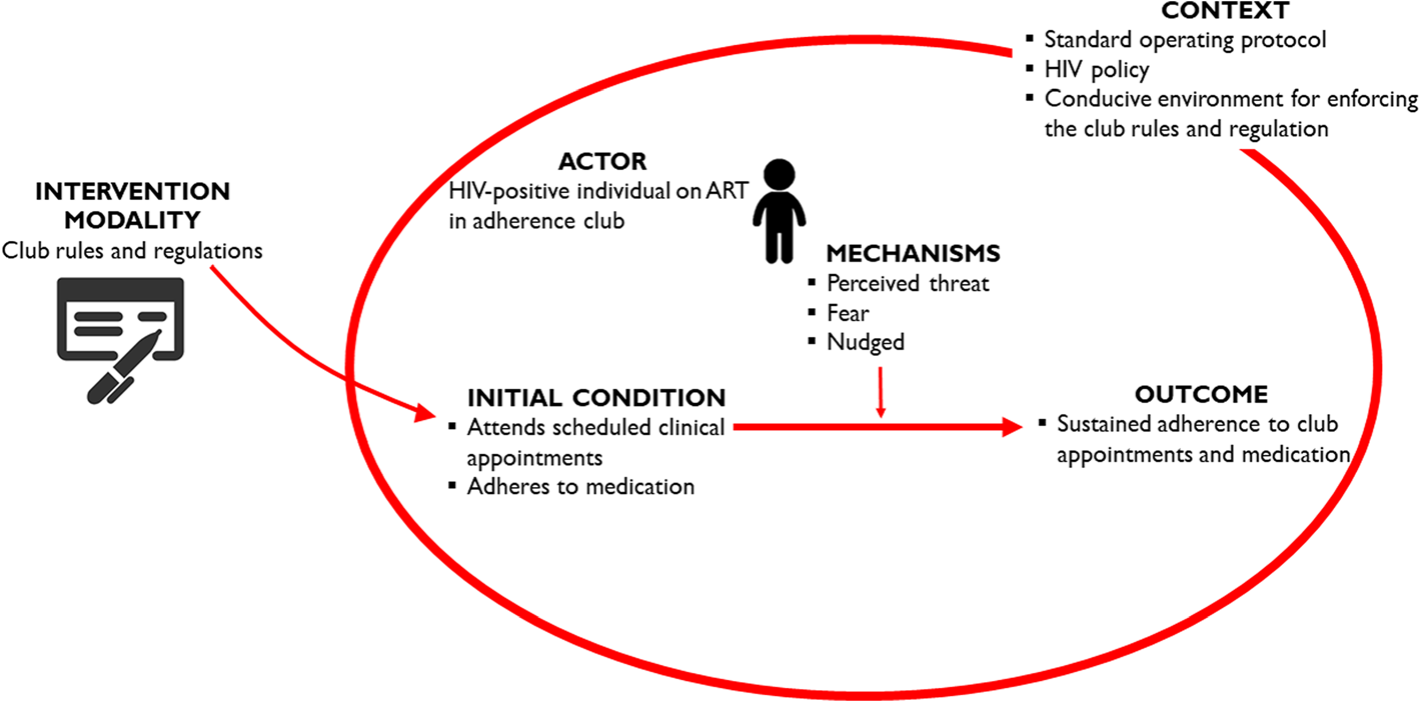
Refined ICAMO configuration in relation to the adherence club rules and regulations

Based on our findings, these rules and regulations introduce mechanisms such as fear (related to losing on the benefits of the club), feelings of being threatened (as they are constantly being reminded of these rules) and feelings of being nudged (told what to do in a positive reinforcing manner) (Fig. 5).

Based on our refined ICAMO configuration in relation to the club rules and regulations, it can be theorised that ***if*** the standard operating rules and regulations of the adherence are applied in the context of the standard operating protocol and HIV policy within a conducive adherence club environment, ***then*** patients are likely to remain in care and adhere to their medication, ***because*** they perceive being threatened, feel nudged or are afraid of losing the club benefits.

### Group formation

The notion of grouping patients with similar clinical characteristics and needs is one of the critical aspects of the adherence club intervention. According to the adherence club programme designers and health managers, grouping patients together for ART stimulates the formation of relationships among the group members in addition to providing easy access to ARV medication.

**Fig. 6:**
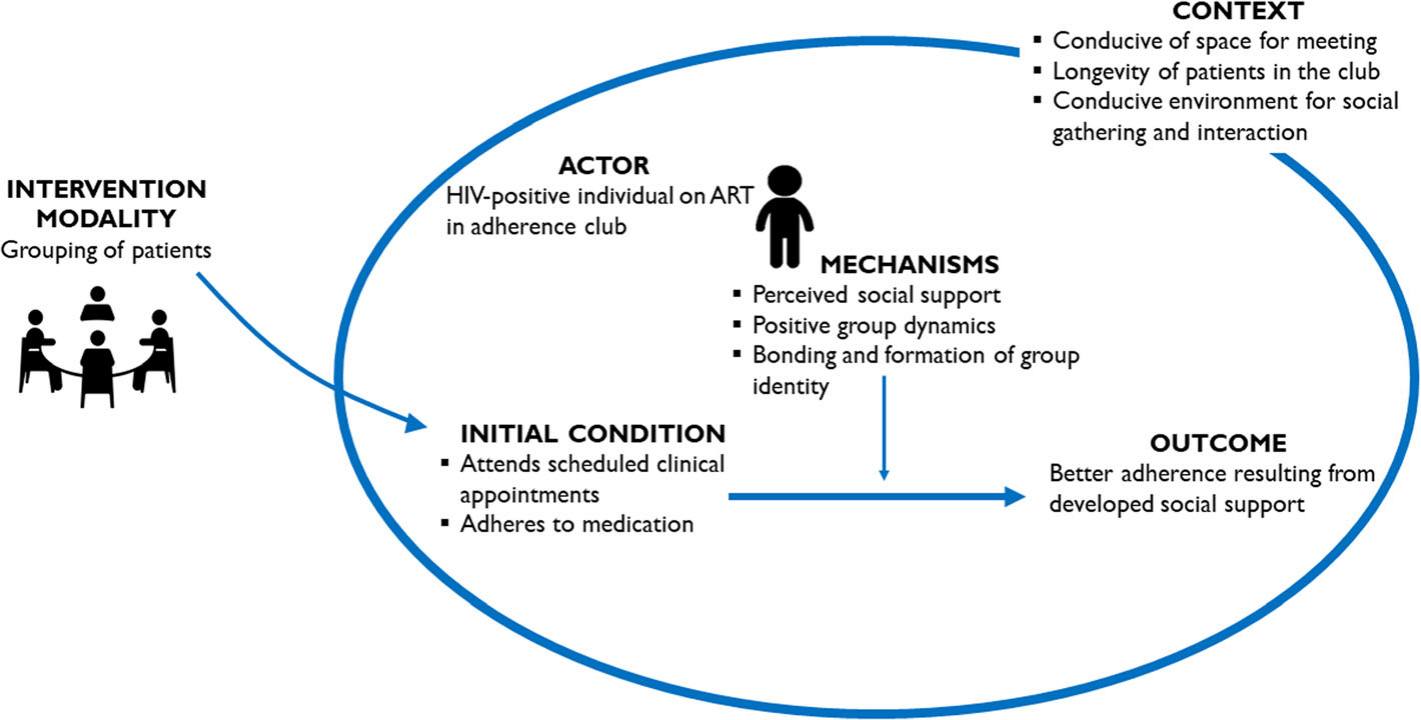
Refined ICAMO configuration in relation to the aspect of grouping the patients

The grouping of patients with a common goal and sharing ‘similar’ experiences engender a new set of mechanisms. Prominent mechanisms provided by the grouping of patients together for targeted care include bonding, which leads to a positive group dynamics and social support. In Fig. 6, the refined ICAMO configuration that was obtained from the cross-case analysis of the three case studies is shown.

Based on this refined ICAMO configuration, the following explanation could be drawn. ***If*** patients are grouped together for targeted ART care in a comfortable meeting space and conducive social environment (away from non-HIV-positive patients), ***then*** they are likely to remain in care and adherence to their medication ***because*** they bond with each other and provide social support to one another.

### Health talks/counselling

Health talks and counselling are among the major secondary activities that take place when patients meet bi-monthly for their medication refill in an adherence club. The club facilitator gives health education talks to the assembled group on relevant topics. These topics most often focus on the challenges that patients face with taking their medication. This opportunity is also used to dispel any doubts, rumours and myths that the patients might have regarding their treatment. The patients are also counselled on the importance of sustained adherence to their medication, and the effects of not taking their treatment as prescribed.

**Fig. 7:**
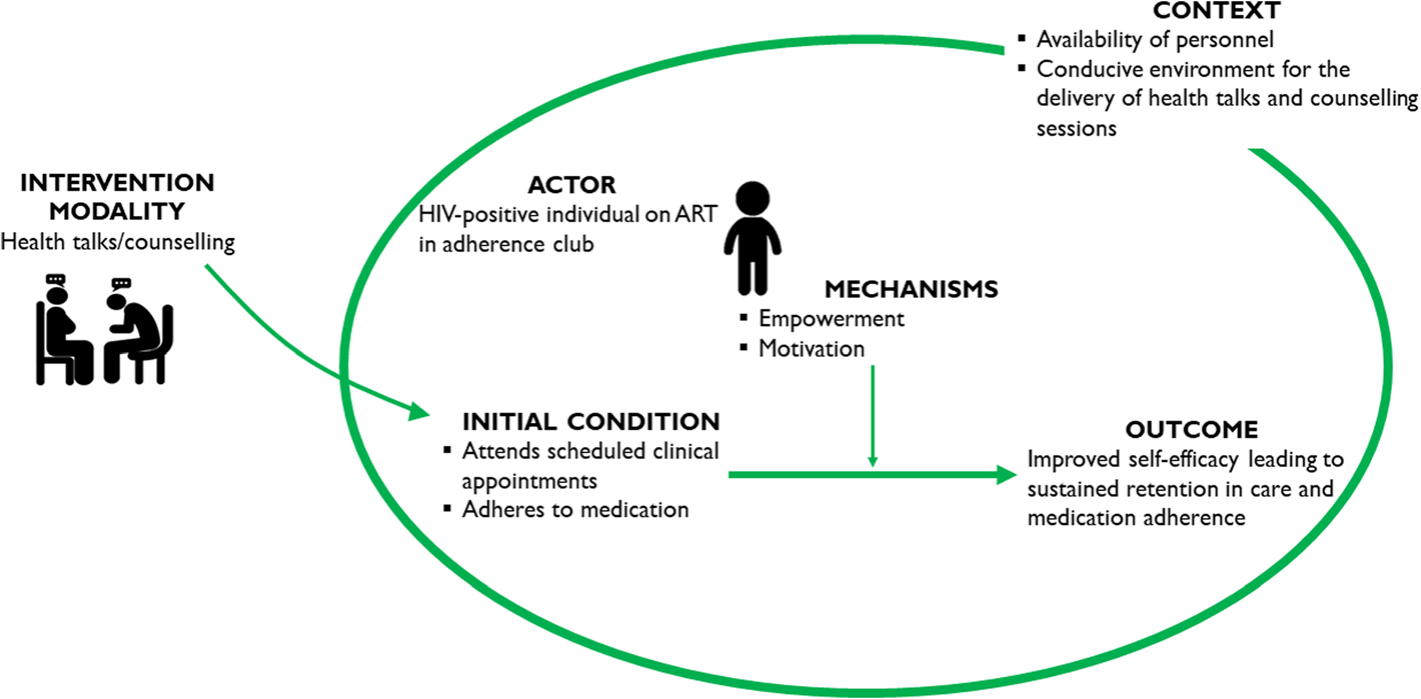
Refined ICAMO configuration in relation to the health talks and education

The adherence club ‘health talks’ and counselling induce distinct sets of mechanism(s) to enhance the treatment and care of patients on ART. Identifiable mechanisms related to health talks and counselling include motivation and empowerment. These mechanisms often translate to a further mechanism, self-efficacy – one’s perception of one’s ability to accomplish a task. In Fig. 7**,** the generative configuration of health talk/counselling provided as part of the adherence club activities is illustrated.

The ICAMO configuration obtained from the synthesis offers the following description. ***If*** grouped patients on ART continuously receive health education and counselling within a conducive environment with available health personnel, ***then*** they are likely to remain in care and continue to adhere to their medication, ***because*** they become motivated and empowered.

### Quick medication pick-up

Quick medication pick-up is a modality of the adherence club intervention that was designed to address the challenge of long waiting times at the clinic when patients come to pick up their medication. The medication is either packed by a Central Dispensing Unit or the clinic pharmacy and made available to the club facilitators to dispense to the patients when they arrive for their club session. In addition to the quick access to their medication when they arrive at the clinic, the widely spaced appointment schedules (2 months) of the adherence club sessions allow patients to get up to 2 month’s supply of their medication.

**Fig. 8:**
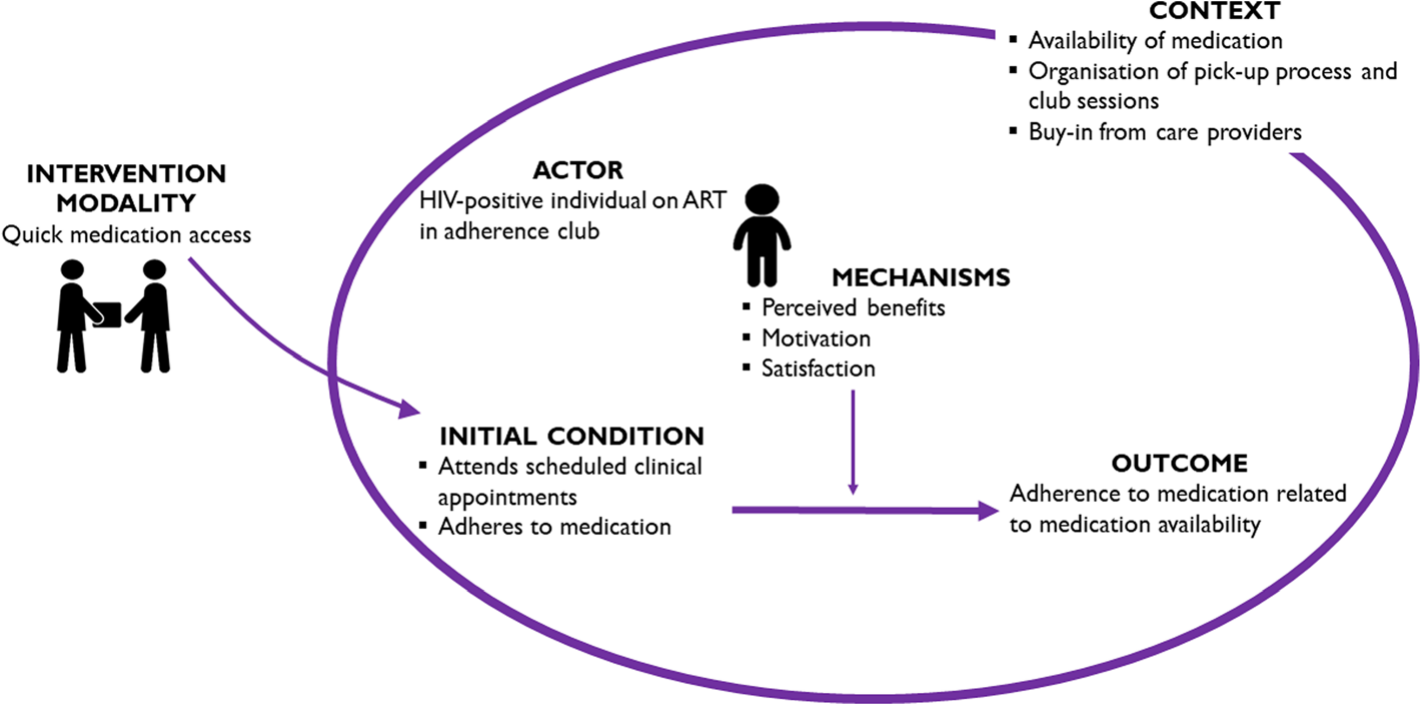
Generative configuration of mechanisms provided by quick medication access

Providing medication to patients through the adherence club with less frequent visits to the clinic introduces mechanisms such as perceived benefit, motivation and satisfaction. How these generative mechanisms produce the intended outcomes of the adherence club intervention is illustrated in Fig. 8.

A refined ICAMO configuration of the quick medication pick-up modality of the adherence club suggests the following theory. ***If*** patients on ART receive quick medication pick-up, ***then*** they are likely to adhere to their medication and remain in care, ***because*** of their perceived benefit, motivation and satisfaction (related to carrying on with other daily activities such as going to work).

### Club facilitator-patient relationship

**Fig. 9:**
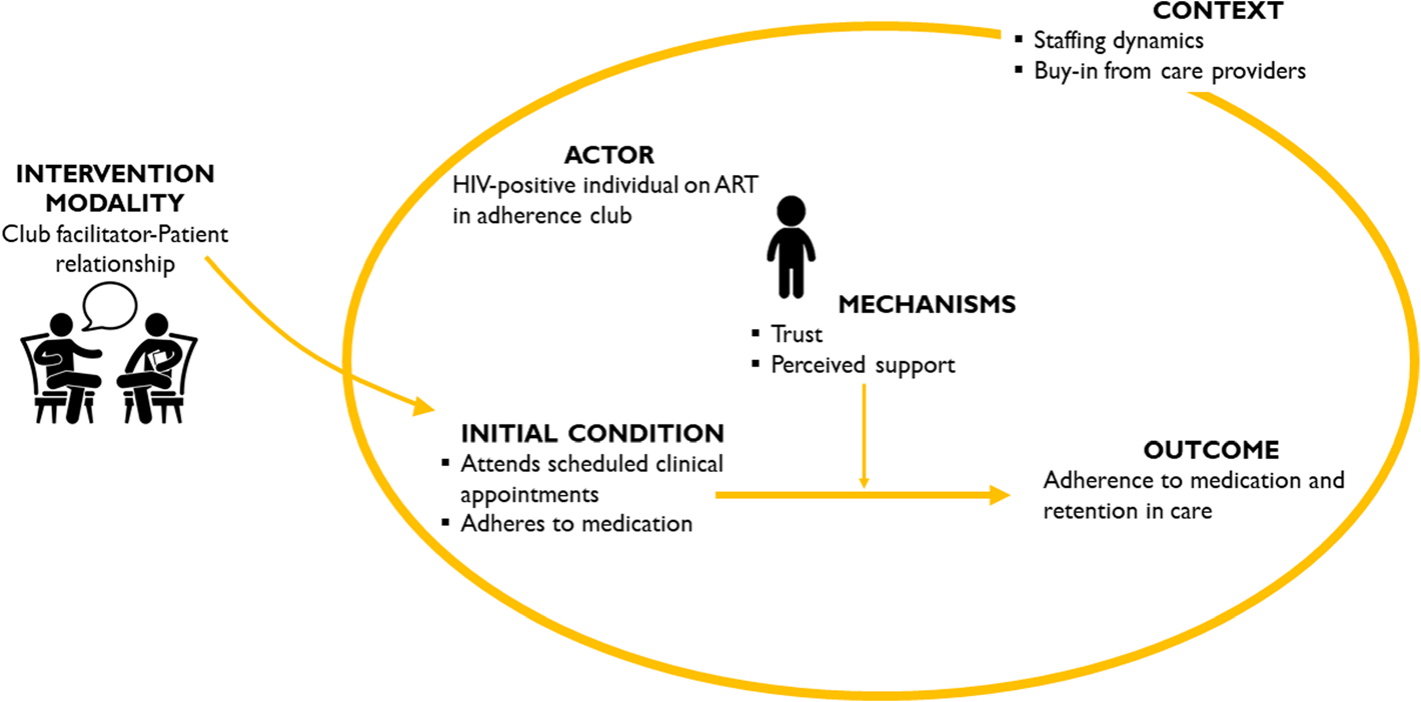
Generative configurations of the mechanisms of the facilitator-patient relationship

The adherence club uses the lowest cadre of the health-care providers to run the activities of the club. The person to do this is usually a peer of the adherence club members with the lowest level of specialisation. The adherence club facilitator prepares and runs the club sessions, makes sure pre-packed medications are available and distributes them to patients, fills in the club register and provides peer education and counselling to the club patients. Therefore, a healthy relationship between the peer club facilitator and the patients presents different sets of social mechanisms to enforce the workings of the clubs. These social mechanisms are ‘trust’ and ‘perceived support’. The generative power of these mechanisms is modelled in the configurational map (Fig. 9).

Although this is not in a strict sense a modality of the adherence club, the relationship shared between the club facilitator and the patients or a group of patients introduces a possible supportive reason of how and why the adherence club works. The refined ICAMO configuration associated with this relation suggests that ***if*** patients receiving ART share a healthy relationship with the club facilitator, ***then*** they are likely to remain in care and adhere to their medication ***because*** they trust the club facilitator and they perceive that the facilitator provides them with social support towards managing their disease.

### Overall intervention

**Fig. 10:**
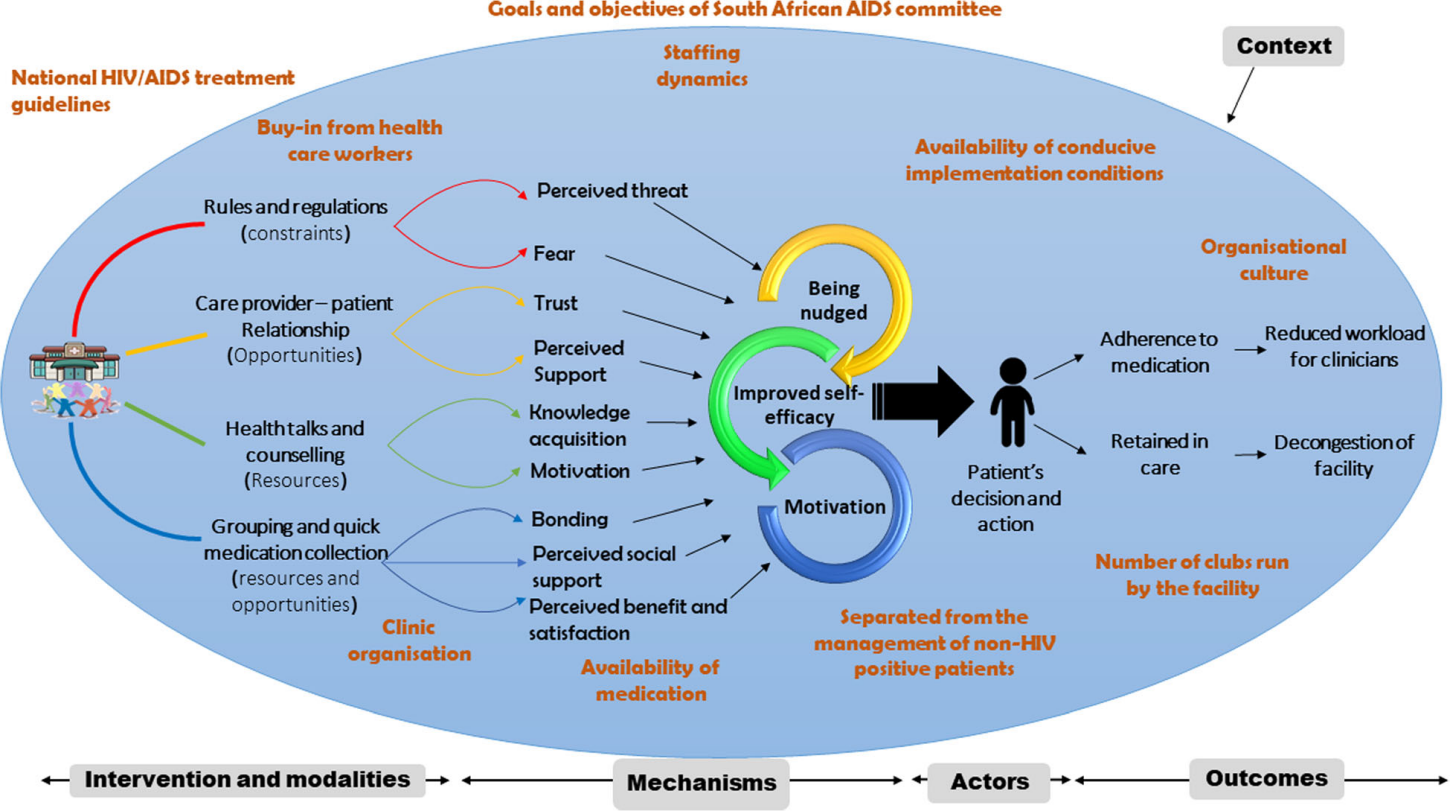
Generative configuration of the adherence club modalities

Byng and colleagues [26] argue that while it is important to have the ICAMO configurations of the different units of the programme, policy or intervention, it adds value to see how these units come together as a whole. They suggested constructing a configurational map to represent the bigger picture. This was achieved by distilling three major mechanisms from the mechanisms identified from the data through the process of abstraction and accentuation (highlighting the most prominent mechanisms) [28]. This follows the logic that certain mechanisms dominate others and occur more frequently and thus become apparent at the level of the actual phenomena in the form of partial regularities, or demi-regularities. The following mechanisms, motivation, empowerment and being nudged were identified. These mechanisms were used to construct a configurational map for the adherence club intervention as an entire intervention with its modalities as illustrated in Fig. 10.

While constructing the bigger picture of how and why the adherence club intervention works, we started by showing how the different components or modalities of the adherence club intervention affect the actors (patients). Then we show how these actors assimilate the impact of the intervention components and through their reasoning, adopt actions and interactions that generate the intended outcomes of the adherence club intervention. Hedstrom and Swedberg [28] describe this approach as micro-macro mechanism model. Based on this generative configuration of the adherence club modalities we constructed the following refined theory.

“Grouping clinically stable patients on antiretroviral therapy **[Actors]** with available resources and buy-in from health-care workers in a convenient space **[Context]** to receive quick and uninterrupted supply of medication, health talks, counselling, immediate access to a clinician when required while guided by rules and regulations **[Intervention]**, works because their self-efficacy improves and they become motivated and nudged **[Mechanisms]** to remain in care and adhere to medication **[Outcome]**”.

It must be acknowledged that for adherence-enhancing interventions to work, the user plays a critical role. Although the treatment and care of ART patients are based on the partnership between the patients and the health-care providers, the major responsibility of the self-care lies with the patients. This responsibility relates to adopting and making use of the resources, opportunities and constraints provided by the adherence-enhancing intervention. Therefore, the participation of the patients is important to obtaining the requisite outcome of the intervention.

## Discussion

In this paper, we aimed to compare different contexts (settings) within which the adherence club has been implemented in the search for a general model explicating how, why and under what circumstances the adherence club intervention works. A confirmatory cross-case analysis was adopted to obtain a refined programme theory. The three case studies were useful in formulating a more refined programme theory of the adherence club intervention. While our initial programme theory (Table 1) suggested that the two theories could independently explain how and why the adherence club intervention works and under what circumstances, testing it in the different case studies illustrated that these two theories operate conjointly to provide a comprehensive explanation, rather than independently or in parallel.

Two of the three case studies (Cases X and Y) were positive cases (confirming the hypotheses of the initial programme theory). That is most of the mechanisms that were hypothesised to ‘cause’ the outcomes were confined within the context of these cases. Case Z was identified as a negative case but it confirmed the initial programme theory in the sense that the absence of the ideal condition(s) identified in the initial programme theory to facilitate the implementation of the adherence club intervention and activate the adherence club programme mechanisms was absent. In fact, the prevailing circumstances had mitigating effects on the implementation of the adherence club programme and deactivated the club intervention mechanisms. This led to a decline in the intended outcomes (poor attendance of club activities and suboptimal adherence to medication) in Case Z.

The transition from case-specific ICAMO configurations to cross-case configurations then to the refined programme theory describes a shift from the specific to a more generalizable theory. This process describes the notion of ‘cumulation’ labelled by Pawson and Tilley [13] that focuses on traversing between the initial programme theory and the theories formulated in the empirical case studies to a refined theory.

Although we obtained a general statement based on the derived ICAMO configurations of how, why, for whom and under what circumstances the adherence club intervention works, we realised in a meeting with the adherence club designers and managers that different patients respond to different aspects or components of the adherence club intervention. We know the adherence club intervention has rules and regulations governing the operation of the five treatment and management components it encompases. The discussion elucidated that some patients respond better to being nudged through the rules and regulations that underpin the functioning of the adherence club rather than being motivated and empowered into remaining in care and adhering to medication through the quick medication pick-up, health education, counselling, and group dynamics. In another differentiated HIV-care model, Community ART Groups, implemented in Tete, Mozambique, it was found that the group members responded more to the ‘Code of conduct’ that bind them, which offered a social control of how the groups functioned [29].

Similarly, different patients respond to different intervention modalities provided by that club intervention depending on their circumstances. For instance, a qualitative evaluation of the Medication Adherence Club intervention in Kenya showed that patients preferred the intervention to the standard treatment scheme because the clubs provided quick access to medication and they had a reduced number of clinic visits, which saved them time and money [30].

Patients using the adherence club intervention in another study identified the quick access to their medication as the main benefit of the adherence club intervention [31]. The authors did not report any evidence of social support taking place among the club members. However, other studies conducted by Dudhia and Kagee [32] and Rasschaert et al. [33] indicated that the patients most valued social support among the members of a group-based adherence intervention.

In a study conducted by Whiteside and Roots [34] to evaluate what works in another differentiated care programme, they identified that the health talk provided to patients and the relationship between the health-care providers and the users were most important to the patients. The authors explained that the health talk enhanced treatment uptake and literacy and the real-time interactions between the patients and care supporters were central to the intervention. They suggested further that the ‘relationship’ provided a conducive psychological environment in which patients received support and encouragement when they experienced stigma, medication side effects and other obstacles to adherence [34].

We believe that because the adherence club intervention has many incorporated treatment and care modalities, there is a chance that it could address the challenges of a wider range of patients using the intervention to enhance their adherence to medication and sustain the attendance of clinic appointments. This assertion is supported by evidence comparing the effectiveness of interventions with multiple strategies (two or more) to single strategies [35]. The findings showed that interventions with multiple components are likely to be more effective compared to those with a single component.

Nevertheless, measuring the effectiveness of complex interventions using outcome-based approaches such as randomised controlled trials poses serious challenges [36]. This is because multi-strategy interventions, for the most part, are complex – having more than one possible outcome, sensitive to context, their implementation depends on the flexibility in tailoring the intervention permitted, and they usually have long causal chains linking intervention with its outcome(s) [37]. To this end, theory-driven approaches to evaluation, such as realist evaluation, have been proposed as alternative methodological approaches that could capture the complexity of these multi-component interventions [38].

Theory-driven evaluations essentially start by developing testable hypotheses of the programme, intervention or policy and testing these hypotheses in identified cases leads to case-specific theories that provide propositions that can be tested and refined [39]. The theories obtained from realist evaluation contribute to “Theories of the Middle Range” as defined by Merton [40]. Such middle-range theories are situated at the level of abstraction that is optimal to be ‘useful’ and ‘testable’. *Middle-range theory involves abstraction, of course, but they are close enough to observed data to be incorporated in propositions that permit empirical testing* [40]. Middle range theories provide explanations that are sufficiently general to explain outcomes across settings and social activities [13].

### Limitations, rigour and trustworthiness

As we moved toward obtaining the refined theory, we recognised that the chances of losing the validity of the data increased. To ensure our data informed our final theory, we referred to the original transcripts to ensure that the ICAMO configurations retained the validity of the interview data. In addition, we organised a feedback meeting with the adherence club programme designers and management at the end of the first two phases (Fig. 1). These feedback sessions were very informative and ensured that we were capturing and representing their ideas appropriately.

The use of positive and negative cases to conduct the cross-case analysis towards formulating the general theory did not only allow us to identify similarities and differences in the cases but to go beyond in supporting or refuting the initial programme theory.

In conducting this study as well as the case studies assembled from the study, we applied all the principles stipulated in the RAMSES II guideline for conducting realist evaluation [11].

## Conclusion

In this study, we set out to conduct a comparative case-study analysis to obtain a more generalizable knowledge about how, why, for whom and under what health systems context the adherence club intervention works or fails to work. Using data from three contrastive sites that have implemented the adherence club intervention; we formulated generative statements using the ICAMO heuristic tool to represent theories from each of the cases. Through cross-case analysis, we formulated a general theory identifying the contextual factors and the mechanisms underlying patients’ practices required to retain them in care and enhance adherence to medication. This theoretical understanding is critical for understanding whether the adherence club intervention has been successful in a particular context, and whether and under what context conditions it can be scaled up or replicated.
